# Construction Notes

**Published:** 1893-02-25

**Authors:** 


### CONSTRUCTION NOTES.

The authorities of the West Fife Diseases Hospital have agreed to proceed with the erection of a kitchen and other necessary outhouses for the equipment of a temporary hospital. This will necessitate the outlay of £90, thus bringing the total cost of the hospital to £600. Messrs. Johnston and Burnie, of Edinburgh, submitted designs and estimates for the permanent hospital, which were ultimately accepted with certain specified modifications.

